# No early gender effects on energetic status and life history in a salmonid

**DOI:** 10.1098/rsos.150441

**Published:** 2015-12-02

**Authors:** Thomas Régnier, Jacques Labonne, Joëlle Chat, Ayaka Yano, Yann Guiguen, Valérie Bolliet

**Affiliations:** 1 INRA, UMR 1224 Ecobiop, Aquapôle, St Pée sur Nivelle 64310, France; 2 Université Pau & Pays Adour, UMR 1224 Ecobiop, UFR Sciences et Techniques Côte Basque, Anglet, France; 3 INRA-UR1037, LPGP, Fish Physiology and Genomics, Rennes 35042, France

**Keywords:** gender effects, energetic status, emergence from gravel, *sdY*, brown trout

## Abstract

Throughout an organism's early development, variations in physiology and behaviours may have long lasting consequences on individual life histories. While a large part of variation in critical life-history transitions remains unexplained, a significant proportion may be caused by early gender effects as part of gender-specific life histories shaped by sexual selection. In this study, we investigated the presence of early gender effects on the timing of emergence from gravel and the energetic status of brown trout (*Salmo trutta*) early stages. To investigate this question, individual measures of emergence timing, metabolic rate and energetic content were coupled for the first time with the use of a recent genetic marker for *sdY* (sexually dimorphic on the Y-chromosome), a master sex-determining gene. Our results show that gender does not influence the energetic content of emerging juveniles or their emergence timing. These findings suggest that gender differences may appear later throughout salmonid life history and that selective pressures associated with the critical period of emergence from gravel may shape early life-history traits similarly in both males and females.

## 1. Introduction

In salmonids, the timing of the first ontogenetic niche shift is a strong determinant of subsequent survival and growth [1]. Previous research has revealed that the timing of emergence is highly variable both among and within clutch [2,3] and is related to individual energetic status (interaction between energy stores and energetic requirements), as individuals with high metabolic requirements emerge earlier with higher energy stores [3]. However, a large part of the variability in emergence timing remains unexplained and further research into the potential causes of such behavioural variations at a critical period of the salmonid life cycle is required. A likely candidate responsible for the observed variability in this key life-history transition is sexual dimorphism on emergence behaviour. In fish, sexual dimorphism accounts for a significant part of intraspecific variation in life-history traits observed in natural populations [4], which can be determined in early ontogeny [5,6].

Sexual dimorphism originates from gender differences in phenotype–fitness relationships (e.g. fecundity, competition, survivorship [4,7–10]) and the proximate cause can be differences in the acquisition, processing and allocation of energetic resources between males and females (e.g. [10]). However, evidence for gender effects on energetic status during early development is scarce in teleosts, and only gender differences in body weight gain were observed in Chinook salmon and Japanese eel larvae [11,12] before sexual differentiation. Sexual size dimorphism has been observed only in juvenile immature masu salmon [5]. These sexual differences, when they arise, are correlated to distinct habitat use and diets [13], which seems to result from differences in energy allocation and requirements [5,13,14]. In maturing or adult fish, sexual dimorphism is well documented. Gender differences in size and age at maturity and reproduction are commonly observed in salmonids [8,10,13,15] and depend on early growth and energy stores [16].

Emergence timing is related to energetic status [3] and conditions subsequent growth [1,6,17] in salmonids, which conditions age at maturity [16]. In addition, emergence corresponds to the onset of sexual differentiation in several salmonids [18,19]. Therefore, early gender effects on energetic status are likely candidates responsible for the observed behavioural variability at this key life-history transition, and may affect the timing of the first ontogenetic niche shift. Such early gender effects may have long lasting effects on traits targeted by sexually antagonistic selection, and lead to sexual dimorphism [20] and the observed variability of reproductive strategies in this genus.

Gender differences in energetic status have been related to the sexual dimorphism throughout ontogeny in salmonids. However, evidence for early development is scarce. This lack of knowledge is a consequence of (i) sexual status being difficult to determine at an early stage in many species and (ii) mechanisms responsible for sex determination being diverse in fish (e.g. genetic, temperature, behaviour [21]), and when sex is genetically determined the genes involved in sex determination are often unknown.

This study investigates early gender effects on energetic status and emergence behaviour in brown trout (*Salmo trutta*). To challenge the hypothesis of early gender differences on energetic status influencing the timing of emergence, recently developed methods that measure individual energetic status in brown trout early stages [3,22] were used. The study also takes advantage of the recent discovery of a conserved master sex-determining gene in salmonids [23,24], which allows the development of accurate molecular sexing approaches [24] that can be applied on early life stages. Using these two approaches make it possible to investigate the presence of early gender effects on a key life-history trait, energetic status, and the potential sex bias in emergence timing, the first critical ontogenetic niche shift in the brown trout life cycle.

## 2. Material and methods

### 2.1 Data

Experimental individuals originate from an artificial crossing with 12 females sampled in the River Bertiz, a tributary of the River Bidassoa in Northern Spain (43.161° N, 1.609° W) on 8 December 2008. On the day of capture, adults were measured for size and weight, their age was determined by scalimetry and eggs were hand stripped and fertilized using sperm from four males (table 1). Adults were returned to the river after the artificial crossing procedure. Eggs were then transported to a hatchery and incubated until 2 February 2009, when 50 larvae from each female were put in separate emergence boxes (four replicates per female) randomly placed in an artificial stream [15]. Emergence boxes were equipped with emergence traps allowing the capture of emerging juveniles each day of the emergence period. Throughout the emergence period, owing to limitations imposed by the experimental device (number of respirometry chambers), up to eight individual emerging juveniles were randomly selected each day, to be measured for metabolic rate (MR) and energetic content using elemental analysis [15] (*N*=157 over the entire emergence period lasting 25 days, between 8 and 18 juveniles per female). Emerging juveniles were kept for 24 h at 11°C (±0.1°C) under dim light for acclimatization and then placed in respirometry chambers of an intermittent flow respirometer [25]. Oxygen consumption (in mm^3^ O_2_) as a proxy for MR was measured over a 24 h period at 11°C (±0.1°C). Because MR is strongly related to body mass, relative MR was used throughout the analysis. Relative MR was expressed as the difference between the observed MR and the expected MR calculated from least-square linear regression of MR on dry weight after double logarithmic transformation. Therefore, fish with higher than expected MR for their weights have positive relative MR and fish with lower than expected MR have negative relative MR [26,27].

**Table 1.** Life-history traits of parental fish.

ID	sex	fork length (mm)	weight (g)	age
A	M	335	425	5+
B	M	290	259	5+
C	M	318	353	6+
D	M	305	296	6+
1	F	274	248	4+
2	F	260	185	4+
3	F	236	162	5+
4	F	280	245	5+
5	F	362	492	4+
6	F	249	199	6+
7	F	254	202	3+
8	F	264	235	6+
9	F	292	303	6+
10	F	500	1254	7+
11	F	326	390	6+
12	F	312	339	7+

After MR measurement, juveniles were measured for size and weight, killed (see Ethics), and dried for 24 h at 60°C. After being placed for 24 h in desiccators over silica gel, dry weight was measured to the nearest 10^−6^ g and juveniles were individually homogenized in an agate mortar for elemental analysis. Elemental analysis was performed with a FlashEA 1112 elemental analyser (Thermo Finnigan, Italy). A sample of 4 mg was collected for each individual in order to determine the percentage of carbon, hydrogen, nitrogen, sulfur and oxygen (CHNSO) per milligram of dry weight. CHNSO composition was then converted to relative energetic content (cal mg^−1^ dry weight) using the formula:

$$\text{Relative energetic content }({\mathrm{cal}\;\mathrm{mg}}^{-1})=\frac{83C+344(H\text{−}0.125O)+25S}{1000},$$

where C, H, O and S refer to the percentages of carbon, hydrogen, oxygen and sulfur in a sample, respectively. After CHNSO analysis, individual samples were kept dehydrated for further genetic analysis as the sex marker was not available at the time of MR and CHNSO analyses.

### 2.2 Genetic analysis

In 2012, genomic DNA was extracted from individual samples used for CHNSO analyses. A first primer pair was designed based on the *Salmo trutta sdY* (sexually dimorphic on the Y-chromosome) male-specific gene (GenBank accession number: JF826019.1), which has been shown to efficiently identify genetic males as this sex-determining gene is only present in males [23,24,28]. It generates a male-specific amplification product of approximately 170 bp DNA in the genus *Salmo*. An additional autosomal amplification product serves as a positive control for the polymerase chain reaction (PCR) amplification. This positive control is derived from the eye-specific lactate dehydrogenase sequence gene (GenBank accession numbers AF488539 and AJ277710) and generates an amplification product of approximately 1 kb. This method can also detect DNA degradation over time, which did not happen in our case. Details concerning multiplex PCR amplification were described in the electronic supplementary material, Supplemental Experimental Procedures. The genotyping assay thus allowed the effective identification of males (two bands) and females (one band).

### 2.3 Statistical analysis

Statistical analyses were performed with the freeware R v. 2.1.1. First, deviation of sex ratio (calculated as the proportion of males in the sample, grouped binary data [29]) from the binomial distribution was tested with a *χ*^2^ goodness-of-fit test, on the residual deviance of a generalized linear model with binomial error and no explanatory variables [29]. Departure from a population-wide sex ratio of 0.5 (balanced sex ratio) was tested using an exact binomial test. Influence of emergence timing on sample sex ratio was then investigated using a generalized linear model with binomial error (and logit link function), sex as the dependent variable and emergence day as the independent variable; each daily data point was weighted by sample size.

Sex effects on individual body mass, body length, energetic content and relative MR were tested using linear mixed models with one of the above listed variables as the dependent variable, sex as a fixed effect and maternal identity as random effect to avoid pseudo-replication. To control for difference in emergence date, emergence timing was used as a covariate in the models (first day of emergence scored as 1). Length was log transformed to satisfy assumptions of linear mixed effects models. Significance of factors in linear mixed effects models was examined by analyses of deviance (type II Wald tests).

## 3. Results

**Figure 1.**
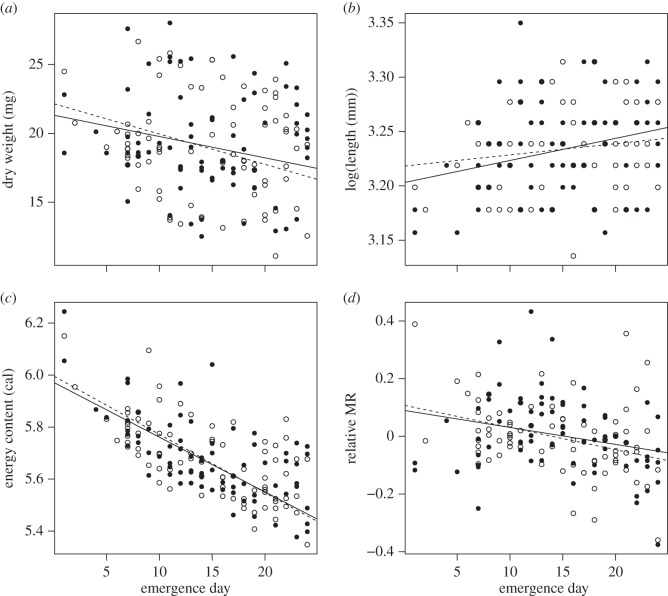
Plots representing the relationships between (*a*) dry weight (mg), (*b*) body length, (*c*) energy content (cal), (*d*) relative MR and emergence day for male and female brown trout juveniles at emergence. Filled circles and solid lines represent females; open symbols and dotted lines represent males. Gender-specific lines are for graphical display only, no gender effects or *gender*×*emergence* day interactions were significant.

Genetic sex typing was successfully performed for all samples except for two individuals due to a lack of sufficient material to perform the analyses. Over the entire emergence period, sex ratio followed a binomial distribution (goodness-of-fit test: *χ*^2^=26.79, 9 d.f., *p*=0.22) and did not depart from equilibrium (exact binomial test: sex *ratio*=0.516, 95%CI: 0.435–0.597, *p*=0.75). In addition, there were no gender differences in emergence timing as sex ratio was not influenced by emergence date (table 2). Gender had no influence on morphological traits as body dry weight (mean (±s.d.) for males: 18.97 (±3.6) mg, for females: 19.26 (±3.82) mg; figure 1*a* and table 2) and body length (mean (±s.d.) for males: 25.32 (±0.99) mm, for females: 25.42 (±1.14) mm; figure 1*b* and table 2) did not differ between males and females at emergence from gravel. Physiological traits were not influenced by gender either, as no differences were observed in relative MR (mean (±s.d.) for males: −0.0039 (±0.13), for females: 0.0042 (±0.13) mg; figure 1*d* and table 2) or mass-specific energy content at emergence from gravel (mean (±s.d.) for males: 5.68 (±0.15) cal.mg dry weight, for females: 5.68 (±0.16) cal.mg dry weight; figure 1*c* and table 2). However, all the traits (dry weight, length, relative MR and energy content) varied with emergence date (table 2).

**Table 2.** (*a*) Logistic regression of sex ratio on emergence day (*n* corresponds to the number of days for which emergent juveniles have been sampled): an odds ratio equal to one indicates that the probability of a male emerging is the same for each value of the considered variable. (*b*) Linear mixed effects models of dry weight, body length energy content and relative MR of different gender throughout the emergence period. *χ*^2^ statistics are type II Wald tests of analyses of deviance, bold *p*-values indicate statistical significance.

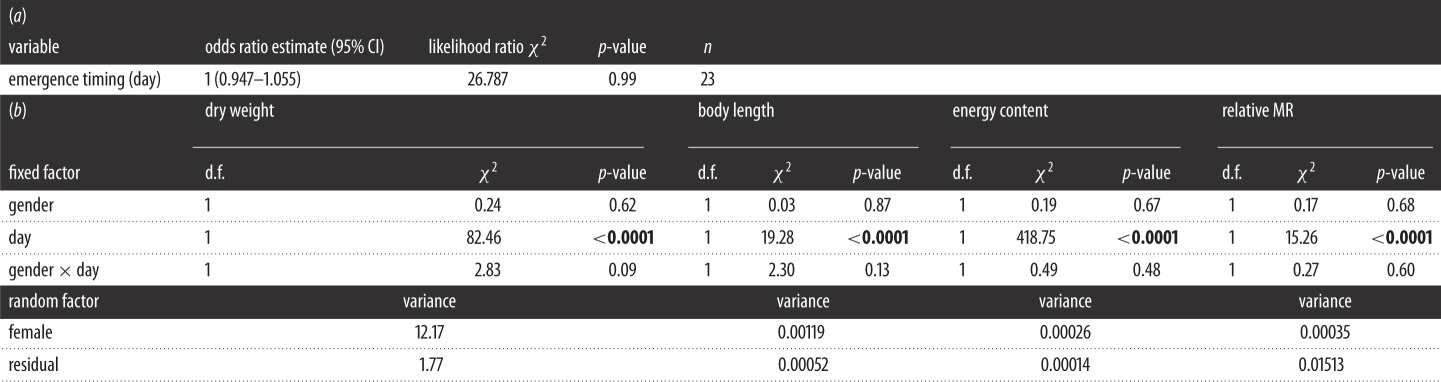

## 4. Discussion

In many previous studies, sex differences have been reported on: (i) the metabolism of closely related salmonids (e.g. *Onchorynchus mykiss*) [30]; (ii) the growth rate of Atlantic salmon (*Salmo salar*) [31,32] and Masou salmon (*Onchorynchus masou*) just after emergence [5]; and (iii) life histories (e.g. sex bias in habitat use, dispersal, seaward migration) of brown trout [13,33] and brook trout (*Salvelinus fontinalis*) [34]. However, to our knowledge, no study has attempted to investigate the footprints of sexual dimorphism as early in the life cycle as in this study. It is worth mentioning that negative results are generally not published often [35], so it is not clear if this lack of information on sexual dimorphism at early stages originates from unpublished negative results (where individual fish could be followed until sexual differentiation), or the lack of investigation, notably due to technical limitations.

In this study, we were able to fill this gap by making use of the recently identified master sex-determining gene, *sdY*, in salmonids [23,24], to track sex differences in brown trout early development. This tool has proved useful to track potential physiological and behavioural sex differences in juvenile brown trout. The results revealed no significant early effect of gender on energetic content, metabolism or emergence timing. In fact, we could not find trends or patterns in the data that suggest that sex has any effect on these traits at that stage.

Therefore, it appears that in the studied population, female brown trout do not allocate their resources differently among sons and daughters as we did not find any difference between the energetic content of males and females at the time of emergence. An alternative hypothesis would be that females provide sons and daughters with different energy stores and that different rate of energy use during incubation leads to a similar energetic state at the time of emergence. However, this last hypothesis is unlikely as no difference in energy use (i.e. MR) has been observed at the time of emergence. Furthermore, because neither energetic status nor emergence timing is influenced by sex, mechanisms that trigger sexual dimorphism during ontogenesis must intervene later in the life cycle.

Despite some clues about early gender differences in gene expression [23], such dimorphism may develop simultaneously with phenotypic sexual differentiation, which starts right after emergence in salmonids (around 800° days in brown trout [18] and brook trout [19]) and, as such, after the period considered in this study. Further studies should address these questions in other salmonids for which *sdY* is expressed during early developmental stages [23]. A similar absence of sex differences on physiology and behaviours at emergence in closely related taxa may confirm that dimorphism occurs later throughout development, when hormonally mediated differentiation takes place.

The lack of early gender differences in the studied brown trout population may result from selective pressures acting on phenotypes during the pre-emergence period affecting both males and females in a similar way (e.g. selection for earlier emergence date, large body size) [1], and as a result may not promote sex differences before emergence from gravel. Alternatively, evidence for early size dimorphism in chinook salmon [11], a species known to display biased sex ratio related to sex differences in survival [36], highlights the requirement for interspecific and inter-population comparisons to understand if environmental gradients that promote gender-specific life histories (e.g. time spent at sea in anadromous populations, intrasexual competition intensity, gender difference in survival) shape early life histories in salmonids through early gender differences in energetic status and behaviour.

## Supplementary Material

1-Supplemental Experimental Procedures

## Data accessibility

Data supporting this article have been uploaded to the Dryad Digital Repository: http://dx.doi.org/10.5061/dryad.8t3nf.
