# Supplementary material for: No early gender effects on energetic status and life history in a salmonid
Source: R Soc Open Sci. 2015 Dec 2;2(12):150441. doi: 10.1098/rsos.150441 (PMC4807450; doi:10.1098/rsos.150441)
Supplement: 1-Supplemental Experimental Procedures [file rsos150441supp1.docx]

**Supplemental Experimental Procedures**

Multiplex PCR amplification was performed in 10 µl using approximately 20 ng of genomic DNA extract, 0.2 mM of each deoxynucleotide, 1.5 mM MgCl_2_, 0.2 µM of each primer (target and control), 0.05 U HotStar *Taq* DNA polymerase and its buffer from QIAGEN. The temperature regime included a first step of denaturation (15’ at 94°C), 30 amplification cycles (1 min at 94°C, 1 min at 52°C, 30 sec at 72°C), and a final extension step of 7 min at 72°C. The primer sequences were as follows: sdY *Salmo* specific primer pairs (forward TGGGCCTATGAATTTCTGAT and reverse ACAGATTTGCGACATGAACA) and positive control (forward GCCAGGAACAAGGTGACAGT and reverse TTGACATGCAGATGGTGTCC). All PCR products were separated by 8% polyacrylamide gel electrophoresis.
